# Reducing Specific Phobia/Fear in Young People with Autism Spectrum Disorders (ASDs) through a Virtual Reality Environment Intervention

**DOI:** 10.1371/journal.pone.0100374

**Published:** 2014-07-02

**Authors:** Morag Maskey, Jessica Lowry, Jacqui Rodgers, Helen McConachie, Jeremy R. Parr

**Affiliations:** 1 Institute of Neuroscience, Newcastle University, Newcastle upon Tyne, England, United Kingdom; 2 Institute of Health and Society, Newcastle University, Newcastle upon Tyne, England, United Kingdom; Tilburg University, Netherlands

## Abstract

Anxiety is common in children with autism spectrum disorders (ASD), with specific fears and phobias one of the most frequent subtypes. Specific fears and phobias can have a serious impact on young people with ASD and their families. In this study we developed and evaluated a unique treatment combining cognitive behaviour therapy (CBT) with graduated exposure in a virtual reality environment (VRE). Nine verbally fluent boys with an ASD diagnosis and no reported learning disability, aged 7 to 13 years old, were recruited. Each had anxiety around a specific situation (e.g. crowded buses) or stimulus (e.g. pigeons). An individualised scene was recreated in our ‘wrap-around’ VRE. In the VRE participants were coached by a psychologist in cognitive and behavioural techniques (e.g. relaxation and breathing exercises) while the exposure to the phobia/fear stimulus was gradually increased as the child felt ready. Each child received four 20–30 minute sessions. After participating in the study, eight of the nine children were able to tackle their phobia situation. Four of the participants completely overcame their phobia. Treatment effects were maintained at 12 months. These results provide evidence that CBT with VRE can be a highly effective treatment for specific phobia/fear for some young people with ASD.

**Trial Registration:**

Controlled-Trials.com ISRCTN58483069.

## Introduction

Anxiety is one of the most common co-morbid conditions in young people with autism spectrum disorders (ASDs), with around half of young people affected [1]. Anxiety disorders are associated with significant social, emotional and economic impact [2] and if untreated can become chronic, with negative effects on other family members [3]. The presence of anxiety symptoms in adolescence is a significant predictor of the development of an anxiety disorder in adulthood [4], indicating the long-term psychological, social and economic significance of childhood anxiety [5]. In ASD, methodologically strong studies have shown specific phobias/fears (for example loud noises, dogs) to be one of the most common anxiety subtypes affecting children across the spectrum [6]–[10].

Mayes and colleagues [11] studied 1033 children with autism (651 with high functioning autism (HFA) and 382 with low functioning autism (LFA)) and found 41% reported to have unusual fears (e.g. toilets, thunderstorms, vacuum cleaners and riding in vehicles including cars, buses and trains). Additional children had common childhood fears and phobias (e.g. fear of dogs, spiders, doctors, sleeping alone etc.); this increased the overall proportion of children who had intense fears and phobias to more than half. These rates did not vary between children with HFA and LFA.

Specific phobias/fears can cause significant distress and have a serious impact on young people with ASD and their families, inhibiting the acquisition of educational or daily life skills [7], [12], and leading to avoidance of everyday situations and reduced participation opportunities. The development of effective interventions to help young people manage anxiety is therefore important, in an attempt to improve longer term outcomes. Early intervention allows young people to develop coping skills, potentially reducing the impact of anxiety on school attendance, academic achievement, social participation and future employment [13].

In recent years, there has been interest in adapting treatments for anxiety for people with ASD, with much of this work based on the principles of cognitive behaviour therapy (CBT). For example, Moree and Davis [14] reviewed the efficacy of modified CBT as a treatment for anxiety in children with ASD. They found four predominant trends in adaptation to make CBT successful for children with ASD: 1. Development of disorder specific hierarchies, 2. Use of concrete visual tactics, 3. Incorporation of child specific interests, 4. Incorporation of parents.

There is some good evidence that CBT can be effective in reducing anxiety for people with ASD, which is particularly important given doubts about whether ASD specific social and cognitive impairments would render CBT inaccessible [15]–[17]. Most of this work has focussed on generalised anxiety and worry or social phobia, with no studies focussing specifically on the use of CBT treatment for specific phobia in ASD. In a recent study, McConachie and colleagues successfully targeted general and social anxiety in a randomised controlled trial of group treatment using CBT principles. Eighty-two per cent of the children, aged 9 to 13 years, recruited through local clinical services had a specific phobia at baseline; a similar proportion continued to have specific phobia following group CBT, suggesting that specific intervention packages are needed to tackle phobia/fears [18].

Graduated exposure is identified as the key therapeutic mechanism in evidence-based treatments for specific phobias and fears [19] but may require adaptation for the particular characteristics of individuals with ASD. For example, graduated exposure may begin with imaginal desensitisation; however, individuals with ASD have difficulties with imagination, which would make producing and controlling imaginal scenes difficult. These difficulties with imagination have been shown in both children [20] and adults [21] with ASD. Additionally, the therapist cannot directly know what the client thinks or is exposed to during the imaginal exposure exercises nor evaluate accurately the level of arousal generated by the phobic stimulus. Those with ASD may also need training in recognising and describing their own feelings.

One way in which traditional approaches to treatment of specific phobias/fears could be adapted to increase accessibility for individuals with ASD is through the use of virtual reality environments (VREs). VREs offer a powerful tool for training as participants become active within a computer generated 3D virtual world. Participants can navigate through an environment which they may find anxiety provoking (for example, a street or school) and interact with objects and people. Newly learned skills can be rehearsed and reinforced in a safe and controlled environment. VREs have been used successfully in the general population to treat fear of flying and heights [22] and fear of public speaking [23]. They have also been used successfully with people with ASD to improve various skills, for example, social understanding [24], understanding facial expressions [25], road safety and fire alarm procedures [26]–[27].

This study was undertaken in collaboration with staff at Third Eye Technologies applying their proprietary immersive technology, ‘Blue Room’, an advanced VRE developed in County Durham http://www.thirdeye.tv.com/. The Blue Room uses audio visual images projected onto the walls and ceilings of a 360 degree seamless screened room. Participants are not required to wear a headset or goggles and can move around the room freely, interacting and navigating through the scenario at their own discretion. Wallace and colleagues [28] carried out testing in the Blue Room and showed that children with ASD feel comfortable in the VRE, and feel they are ‘present’ in the scenarios depicted. In this development study we investigated whether a combination treatment using a CBT approach with a ‘wrap-around’ or ‘immersive’ VRE reduced specific phobia or fear for young people with ASD, and whether this would lead to functional improvements in managing real life anxiety provoking situations.

## Methods

The protocol for this trial and supporting TREND checklist are available as supporting information; see Protocol S1 and Checklist S1.

### Participants

**Figure 1:**
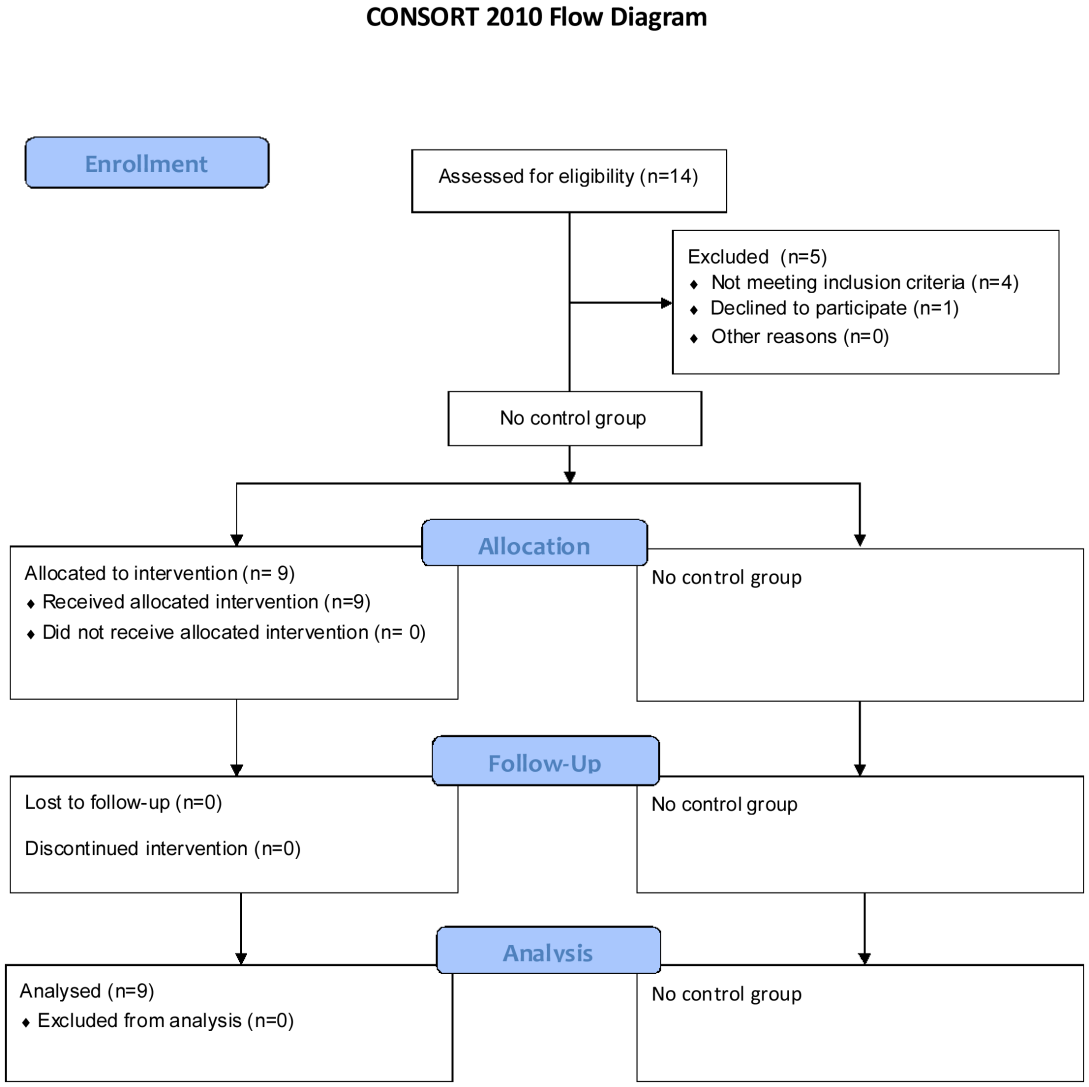
CONSORT flow diagram.

Nine verbally fluent boys with a clinical ASD diagnosis and no reported learning disability, aged 7 to 13 years old, were recruited. Recruitment was from the database of children with autism spectrum disorder living in the north east (Dasl^n^e) [29]–[30], held at Newcastle University (8 participants) and through a local Community Mental health team (1 participant); see Consort Flow Diagram (Figure 1). Recruitment from the database was via mail out to all young people within the age range who lived within the vicinity of the VRE. At the time of joining the database parents provide details of their child’s clinical ASD diagnosis and this was independently corroborated by the local multidisciplinary team clinicians who made that diagnosis. Clinical multidisciplinary teams in the North East of England follow best practice as laid down in guidelines from the UK National Institute for Health and Clinical Excellence [31].

All parents completed the Social Communication Questionnaire (SCQ) [32]; all children’s scores were compatible with an ASD diagnosis (range 13 to 31) [33]. All children had a specific phobia/fear for which the Third Eye team were able to create a visual scenario (see Table 1). One child was on medication (Fluoxetine for anxiety).

**Table 1:** Presenting phobia/fear, VRE scene designed and outcomes following treatment (functional progress with phobia/fear, and Target Behaviour Scores).

Child	Presentingphobia/fear	VRE scenedesigned	Outcome following VREtreatment sessions;examples showing outcomesat 6 weeks, 6 monthsand 12–16 months	Post treatmentTarget BehaviourScores		
				6weeks	6months	12–16months
**A**	Afraid to go near busy roads in casehis younger sister or dog ran in theroad. This led to a refusal to crossbusy roads.	Roadside scene where numberof cars gradually increase anda dog ran alongside the road	At 6 weeks A was able to walk alongsidea busy road although still anxious aboutcrossing. At 6 months and 12–16 monthshe remained anxious while walkingalongside and crossing a busy road. Hewas able to walk alongside andcross a quiet road.	3	3	3
**B**	Pigeon phobia. Participant could notsit near a window in case a pigeonflew past and became very anxiouswhen going to his local town wherehe knew pigeons would be present.	Playground scene where numberof pigeons gradually increased.	At 6 weeks, B was able to control his anxietywhen encountering pigeons by using thetechniques he learnt in the VRE. He wasable to sit by windows and walk pastpigeons. At 6 months post treatmenthe continued to use the techniqueslearned to control anxiety. At 12months post treatment he was ableto manage his anxiety withoutthe need for relaxation exercises	2	2	1.5
**C**	Shopping/social anxiety resulting inchild walking behind parents whenshopping with his hood up andrefusing to speak to even peoplehe knew.	Petrol station kiosk whereparticipant ‘picked up’ anewspaper and graduallybuilt up a four part conversationwith avatar	At 6 weeks, C was able to buy anewspaper at a local shop withhis parents waiting outside. Atsix months post treatment hehad progressed to shoppingindependently with friends. At oneyear he remained able toshop independently	1	1	1
**D**	Afraid to get on a crowded bus. Thefamily were reliant on publictransport but child would refuseto get on crowded bus and familywould regularly have to wait for30–40 minutes for uncrowdedbus to arrive.	Bus stop at which a bus arrivesand participant virtually gets onand the number of people onthe bus is gradually increased	At 6 weeks, D was able to geton a crowded bus. At 6 monthspost treatment, D was able toget on crowded buses and alsocrowded trains. At one yearpost treatment the family reportedthey hardly ever thinkof his phobia nowadays	1	1	1
**E**	Shopping/social anxiety. Childwould refuse to speak to shopassistant and become highlyanxious in supermarkets andshops.	Supermarket kiosk whereparticipant choose sweets,went up to the counter andtalked with an avatar shopassistant	At 6 weeks, E was able to go toreal supermarket kiosk, buysweets and respond to shopassistant with a family member inthe background. He remained ableto do this at 6 months and at 12months post treatment couldachieve the same with a supportworker in place of family member.	2	1.5	1.5
**F**	Crowded buses. Child wouldrefuse to get on any bus in case‘strange people’ got on. Familywere concerned as he wouldshortly be changing to a schoolwhich required a bus journey.	Same scene used as for previousparticipant with this phobia	At 6 weeks and 6 months, F wasable to walk to the bus stopbut there was no improvementin his fear of getting on buses.At 12–16 months he got on apublic bus with support fromhis family but this was associatedwith high anxiety	5	5	4.5
**G**	Being a passenger in a car.Following an accident whenbeing driven by his grandmother,participant refused to get in a carwith a female driver.	Car that participant virtually gotin to and was the passengeras car travelled through acity scene	After two VRE treatment sessions,G was able to be a passenger in acar with a female driver. At 6 weeks,6 months and 12–16 monthsthis improvement was maintained	1	1	1
**H**	Afraid to cross a bridge(particularly if waterunderneath) and afraid ofheights in general e.g.escalators and stairs.Participant would oftenrefuse to cross bridges,climb stairs etc.	Bridge scene where we couldgradually increase the heightof the bridge and the waterunderneath	At 6 weeks, H was able to cross abridge. At six months he transferredskills learned to other height situationse.g. escalators and multi-storey carpark stairs. At 12–16 months thefamily no longer thought abouthis previous phobia as he was ableto tackle all the height situationsthat previously worried him	1	1	1
**I**	Speaking in class.Participant would notraise hand orcontribute in class,even when he knewthe answer.	Virtual classroom wheregradually increase thenumber of children and theavatar teacher asks 5 questions(questions progressively requiring moredetailed answers)	At 6 weeks, child I was able to answerquestions in the subject class hewas most confident in (maths).At six months he had started raisinghis hand and answering questionsin English class. By 12–16months he had progressed to answeringquestions in less favoured subjects.	2	1.5	1.5

Target behaviour scoring: 1 = normal; 2 = markedly improved; 3 = definitely improved; 4 = equivocally improved; 5 = no change; 6 = equivocally worse, etc. Those with scores of 3 or less are classified as responders to a treatment.

### Ethics Statement

A positive Ethics opinion and approval of the study was given by Sunderland NHS Research Ethics Committee (reference 12/NE/0018).

### Research procedure and measures

After receipt of an Expression of Interest, MM met with the young person and their parents in their home to explain the study and show video clips of the Blue Room VRE, answer any questions and obtain informed written consent and assent from both the child and their parents/guardians. This was documented on a parent/guardian consent form and a young person’s consent form. This written consent procedure was approved by Sunderland NHS Research Ethics Committee. Baseline questionnaires (see below) were also undertaken with parent and child. The parent(s)/guardian(s) of all individuals described in case studies and/or in photographs gave written informed consent (as outlined in PLOS consent form) to publish case details.

Each child then had one assessment and preparation session at home (lasting approximately one hour), and then four VRE exposure sessions (2 sets of 2 sessions lasting 20–30 minutes each). Approximately two weeks after the final VRE session, MM visited the family and obtained confidence ratings and a verbal report from the family as the child tackled the real life target situation.

Six weeks, six months and 12–16 months after the final VRE session MM contacted the family to repeat anxiety questionnaires and obtain further verbal report about management of the target anxiety situation with parent and child.

### Measures

#### Pre and Post Treatment

*Spence Children’s Anxiety Scale-parent version (SCAS-P) and child version (SCAS-C):* The SCAS [34] was developed to assess anxiety symptoms in children in the general population. The SCAS-C has 44 items on a 0 (never) to 3 (always) scale and comprises six subscales, including panic attack and agoraphobia, separation anxiety disorder, social phobia, physical injury fears, obsessive compulsive disorder and generalized anxiety disorder. Six items are positively worded filler items (excluded from the parent version). The measure is widely used in ASD studies [35], [15], [18], with good criterion validity in typically developing children (high correlation with the Anxiety Disorders Interview Schedule) [36]. The SCAS shows high internal consistency, for the total scale, and each subscale [34] and good validity, for example, distinguishing between groups of typically developing children with and without anxiety disorder [37].

*Target Behaviours:* Standardized scales may not include the exact item(s) of most concern to the participant or their caregivers, and may fail to reflect real change important to the individual family [38]. Target behaviours were used to record change over time for anxiety relating to a specific situation. The protocol used was developed by The Research Units on Paediatric Psychopharmacology and involves working with families to identify one or two problem/target behaviours. Identifying target behaviours includes asking questions such as ‘how often?’, ‘how distressed?’ and ‘how does it interfere with daily activities?’ asked in a standard format to the parent and child, to enable a vignette to be written about behaviours and their severity/impact [35]. In our study, vignettes from baseline and 6 weeks, six months and 12–16 months after the end of treatment were compared by an expert panel to assess the degree of change of target behaviour from baseline on a 9 point scale, from ‘normalised’ (1 on scale) to ‘disastrously worse’ (9 on scale). Those whose paired vignettes were rated 3 or less (corresponding to ‘definitely improved’ or better) were classed as responders to treatment.

#### During treatment

**Figure 2:**
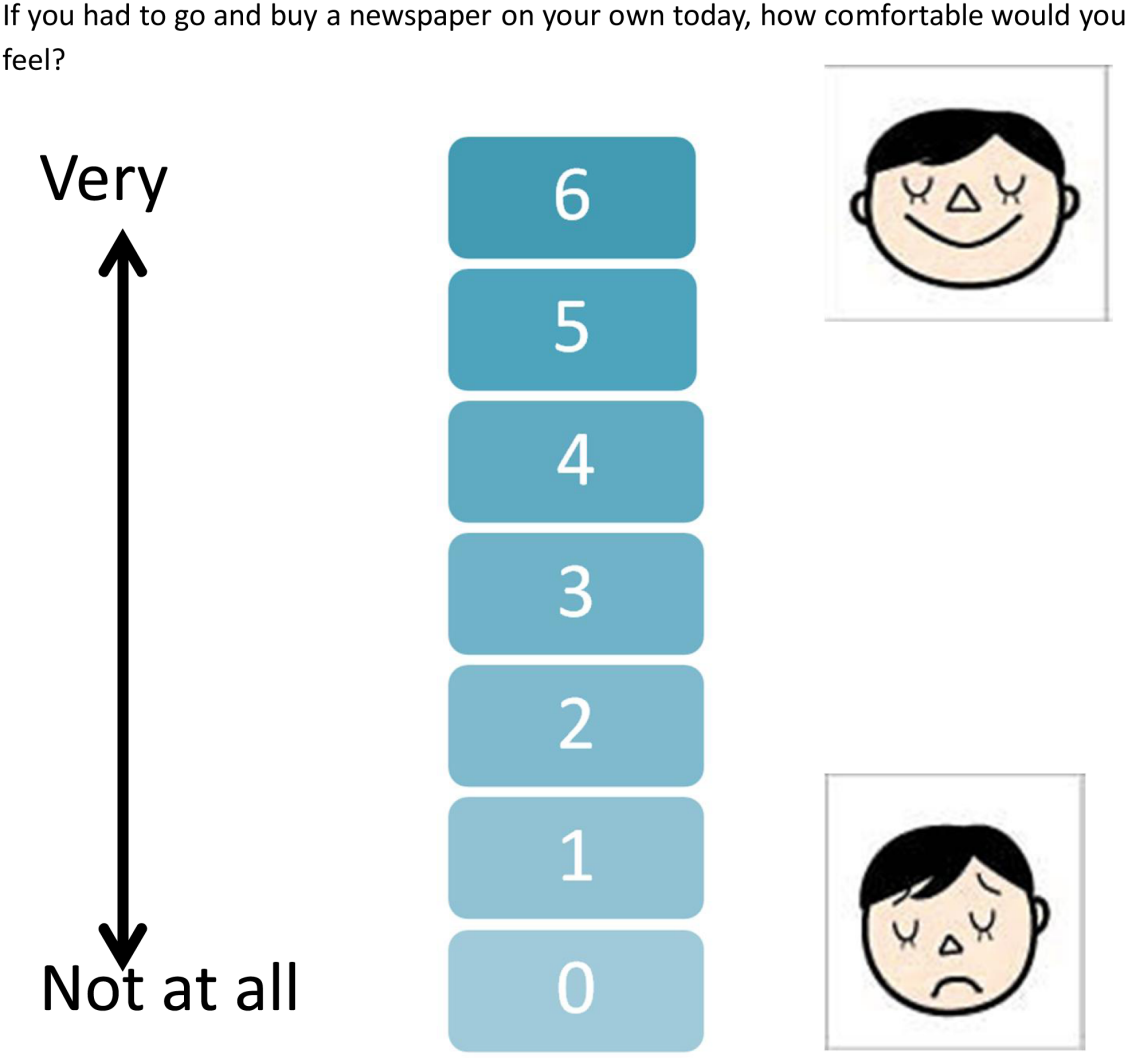
Confidence rating scale for shopping scene (child’s version).

Confidence ratings: Children rated their confidence at tackling their target situation at the beginning and end of each of the VRE sessions, and at a subsequent ‘real life’ occasion. Parents also rated their perception of the child’s confidence at the beginning and end of each of the VRE sessions. Ratings were from 0 (not at all comfortable) to 6 (very comfortable). Examples of confidence rating scales for a shopping scene are shown in Figure 2 (child’s scale).

### Treatment procedures

The first home visit was conducted by MM and a psychology assistant (JL); during this visit the team discussed with the child and parents the selection of the specific phobia/fear target to be addressed in the VRE. The psychology assistant (who the child knew would be with them during the VRE sessions) explored with the child possible steps to full exposure, including starting with short Blue Room VRE sessions and increasing the length of exposure (for example being the passenger in a car), or increasing aspects of the scene (such as additional people in a supermarket or classroom). The psychology assistant explained the ‘safe’ nature of the Blue Room, and that reducing or stopping exposure was possible, and under the child’s control.

Basic cognitive and behavioural therapy techniques were introduced. These included identifying feelings (how different parts of the body feel, what thoughts the child may have), and introducing the concept of a ‘feeling thermometer’, a visual analogue scale used to communicate level of anxiety, using the child’s own word for what they felt. During this visit there was approximately 45 minutes training in techniques such as relaxation, deep breathing, and using positive coping thoughts when experiencing anxiety. This initial training was consolidated in the Blue Room sessions.

There was then a break of several weeks while staff at the Blue Room prepared the specific VRE scenes for the child. Each scene was individualized and took approximately four days of programming time, although this input decreased when scenes were repeated (e.g. shopping, crowded buses).

Each participant then received four 20–30 minute treatment sessions in the Blue Room, over two half days. The two sessions on one day were broken up by a 15 minute break. The second visit was usually scheduled for several days later. Therapy in the Blue Room was delivered by the psychology assistant, overseen by a consultant clinical psychologist.

**Figure 3:**
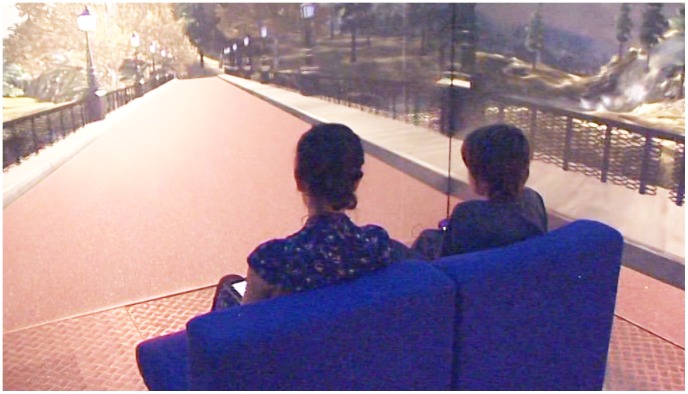
Young person in the Blue Room.

At the beginning of each VRE treatment session, a relaxation scene with a soothing soundtrack (e.g. swimming dolphins) was played for several minutes. The therapist coached the child through breathing and stretching exercises during this time. When the child felt ready, the therapist began the target virtual scene (operated by an iPad controller) (Figure 3).

The scene always began at a challenge level for which the child rated low anxiety on the ‘feeling thermometer’. For example, for a child afraid of shopping the therapist would begin with simply going into an empty shop and taking something to the counter. This would be repeated several times as required by the child, with the therapist checking how the child rated their anxiety on the visual anxiety scale, how their body was feeling and what they were thinking. This was an opportunity to make the children aware that they could reduce the sensations in their body through breathing and stretching exercises, and that they could challenge the thoughts that occurred in the situation and replace these thoughts with more confident statements. The scene was gradually increased in challenge over the four sessions but with the same checking in process by the therapist at all stages. For example, in the shopping scene the length of the verbal exchange with the virtual shop assistant was increased, but at a rate that enabled the child always to feel comfortable and relaxed.

**Figure 4:**
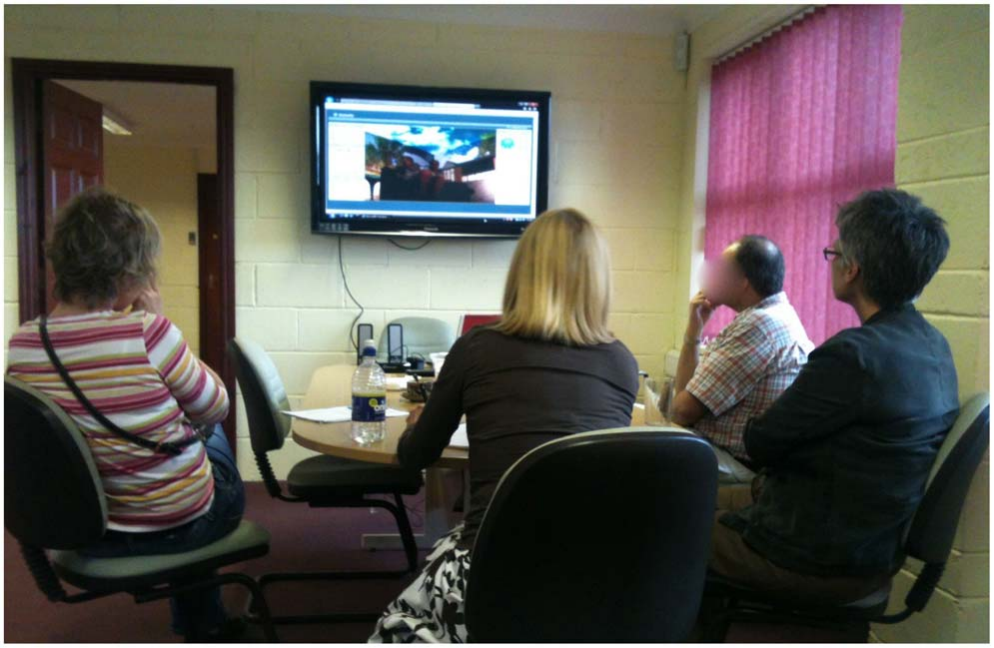
Parents observing child in Blue Room via video link.

While the child was in the Blue Room, their parent(s) were able to observe the session via video link in an adjacent room (Figure 4).

Parents were able to watch the therapist interacting with their child and learn the techniques she was using. They were also able to observe their child cope with increasing levels of challenge and succeed.

At the end of the final Blue Room session, each family planned with the psychology assistant how to gradually increase exposure to the real life specific phobia/fear situation. For example, for children afraid of shopping, parents were instructed to gradually withdraw their support within the situation, and encourage their child to increase the verbal exchange with a shop assistant.

### Analysis

Target behaviour vignettes and functional ability in the specific phobia/fear situation were compared for all time points.

The mean and standard deviation for the SCAS total scores for the group at each time point were calculated. The Reliable Change Index (RCI) [39] was calculated for each of the participant’s total SCAS scores at 12–16 months after the VRE treatment sessions relative to the total score at baseline. We also report the RCI for the group results at 12–16 months post intervention. The RCI is used to determine whether the magnitude of the change in an individual’s score before and after an intervention is statistically reliable (RCI of ≥1.96 is statistically significant). To calculate the RCI, Cronbach’s alpha was calculated from archival data from a previous study of 111 children with ASD aged between 8–16 years (α = .93) and 232 parents of children with ASD (α = .94) (Rodgers, personal communication, unpublished data). The Leeds Reliable Change Indicator [40] was used for our analysis. This has been designed for clinical research studies and provides a calculation of whether change in scores reported by a client at the end of treatment (in comparison to baseline) is clinically significant.

## Results

Table 1 shows each of the nine children’s phobia/fear situation, the scene which was designed for them, the rated change in target behaviours after the VRE treatment sessions, and the pre and post treatment functional ability in the specific phobia/fear situation.

### Target behaviour ratings

All children with the exception of child F improved from their baseline ability to handle the situation they worked on in the VRE. Eight out of the nine children were responders to the treatment, although one of the children (child A) improved less than the other responders. Four of the children (Children C, D, G and H) completely overcame their phobia/fear and normalised (target behaviour rating 1); these effects were maintained six months, and 12–16 months post treatment (Table 1).

### Confidence ratings

**Figure 5:**
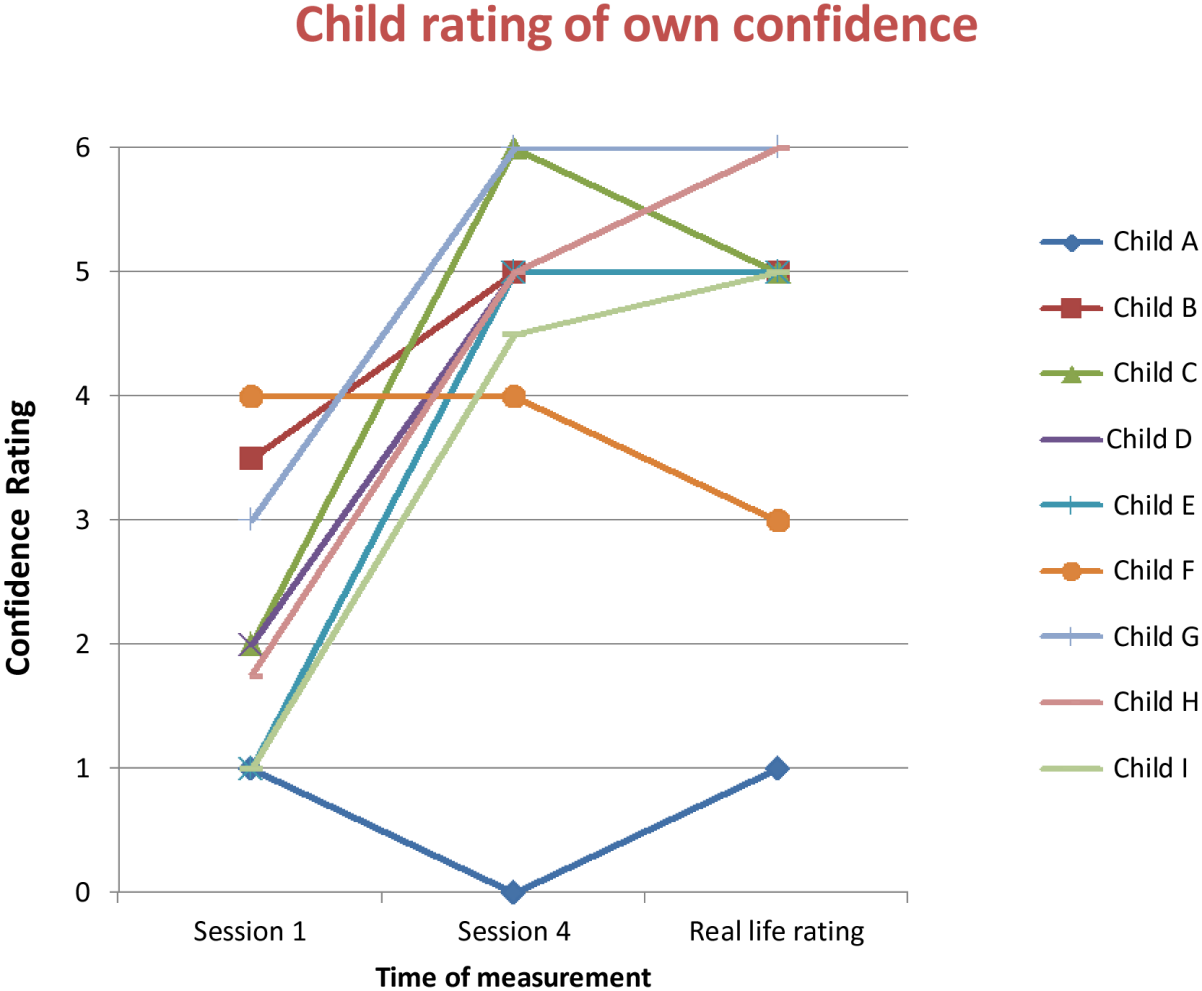
Change in confidence levels as rated by participants.

**Figure 6:**
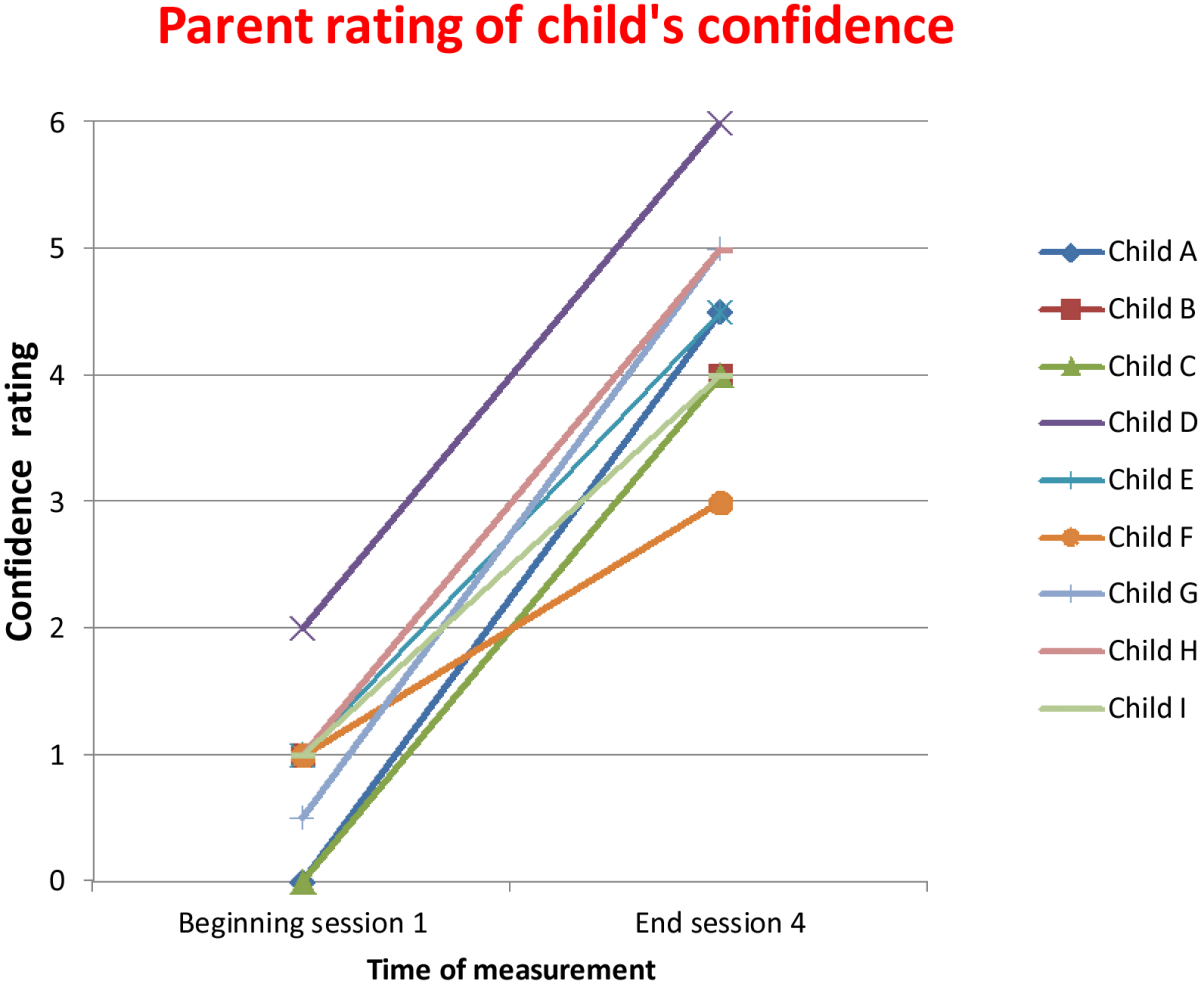
Change in confidence levels as rated by parents.

Figures 5 and 6 show the change in confidence in tackling the target situation over the course of the sessions (and in real life for the children), as rated by the children and the parents.

For seven out of nine of the children confidence ratings improved from before treatment to the end of session 4. This improvement in confidence was maintained at two weeks post VRE sessions when the researcher observed the family while they tackled the real life sessions.

### Spence children’s anxiety scale

The total scores for the SCAS for both the parent and child versions and the RCI at 12–16 months post treatment are presented in Table 2.

**Table 2:** Parent (SCAS-P) and Child (SCAS-C) reported Spence Children’s Anxiety Scale total scores (all RCI comparisons with pre-treatment scores).

	Meanage (SD)	Pre-treatment		Six weekspost treatment		Six monthspost treatment		12–16 monthspost treatment		Reliable ChangeIndex Pre-treatment - 12–16months post treatment	
		SCAS-P	SCAS-C	SCAS-P	SCAS-C	SCAS-P	SCAS-C	SCAS-P	SCAS-C	SCAS-P	SCAS-C
Mean (SD) wholeSample (n = 9)	11.2 (2.0)	40.7 (14.3)	43.3 (15.0)	34.2 (17.9)	35.5 (21.8)	31.1 (15.9)	29.6 (19.7)	29.7 (19.8)	27.9 (20.1)	1.6	1.94
**Individual data**											
**Child**	**Age (years)**	**SCAS-P**	**SCAS-C**	**SCAS-P**	**SCAS-C**	**SCAS-P**	**SCAS-C**	**SCAS-P**	**SCAS-C**	**SCAS-P**	**SCAS-C**
A	10	65	67	63	75	58	58	72	50	–1.02	2.26*
B	12	38	43	31	37	24	19	19	18	2.77*	3.32*
C	13	43	48	32	34	31	33	31	31	1.75	2.26*
D	10	26	+	24	+	20	+	21	+	0.73	+
E	12	37	49	46	31	35	NA	26	NA	1.6	NA
F	11	63	51	58	51	54	44	50	54	1.9*	–0.4
G	7	27	43	26	39	30	40	26	33	–0.15	1.33
H	13	35	18	11	13	12	10	9	9	3.79*	1.2
I	13	32	27	17	4	16	3	13	0	2.77*	3.59*

+ = child unable to complete SCAS due to receptive language level.

NA = child refused to complete.

*If RCI is 1.96 or more, the change is statistically significant.

For males aged 8–11 years, a total score above 40 indicates levels of anxiety above the normal range for that age group. For those aged 12–15, a total SCAS score above 33 indicates levels of anxiety above the normal range. Therefore child A, B, C, E and F had elevated levels of anxiety at baseline on both the parent and child questionnaires, as well as child G on the child SCAS and child H on the parent SCAS.

At six months after treatment child A and F continued to have elevated scores on both the parent and child SCAS, and child E continued to have elevated scores on the parent SCAS. All the other children scored within the normal range for their age group. Therefore, post treatment child B, C, G and H had anxiety scores within the normal range.

At 12–16 months after treatment child A and child F continued to have elevated scores on both the parent and child SCAS. Child E’s scores had improved and were within the normal range. All other children scored within the normal range for both the parent and child SCAS.

The major change for the majority of the children in this study was their functional ability to handle the real life situation they were previously afraid of. Nevertheless, for some children there was also reliable change in the SCAS total values by 12–16 months post VRE treatment for both parent reported SCAS (children B, F, H and I) and child reported SCAS (A, B, C, and I). It was not the intention of the current study to be powered to undertake group based analysis of results –although this will be the intention of the design of the next study which will have a larger sample size. Nevertheless, the RCI values for the group results at 12–16 months also show RCI values approaching reliable change.

In summary, eight of the nine children benefited from the VRE treatment, and went on to tackle their target situation in real life, within six weeks of the final VRE session; four children completely overcame their phobia. At 6 months and 12–16 months post treatment, the improvement in target behaviours was maintained or improved for all children.

## Case Descriptions

Three representative cases are described in more detail below.

### Child D

Child D was a 9 year old boy with fear of crowded buses and underground trains. The family had no car and were dependent on public transport. D had tantrums on a crowded bus or refused to get onto an approaching crowded bus. This often led the family to wait 30–40 minutes until a much less crowded bus came along, or not go out as a family.

Before the first VRE session, D rated himself as having low confidence, 2 (on a scale of 0 to 6); his parent rating was also 2. A VRE scene was designed where D stood at a virtual bus stop, waited for a bus and then virtually got onto it, and found a seat. The number of people on the approaching bus increased over the four sessions.

At the end of the final VRE session, D rated himself as confident in this scenario (6, with parent rating 5). This confidence translated into functional improvement; the family reported that immediately after the final VRE session, he was able to get onto a crowded bus. Two weeks later, D and his family were able to board any bus, irrespective of the number of people on it, and after six weeks, D was able to ride on crowded underground trains. At six months after VRE exposure, this positive effect was maintained with. At 12–16 months after VRE exposure this situation was maintained with D actively enjoying outings on public transport. D’s mother commented ‘D has not looked back since visiting the Blue Room and never worries about crowded buses and trains anymore’.

### Child B

Child B was a 12 year old with a phobia of pigeons. B could not sit by a window in his own house and panicked if faced with pigeons while outside. Going out was preceded by considerable anxiety about encountering pigeons, leading to significant impact on the family’s participation in outside events.

A VRE playground scene was developed, initially including one pigeon and then building up over 4 sessions to exposure to 10 pigeons. The pigeons flew in and out of the VRE, with realistic accompanying sounds (which were part of B’s phobia). The VRE allowed B to virtually move toward and away from the pigeons. Eventually B could tolerate 10 pigeons flying in, and virtually ‘walk’ past them, before they flew out again. Before the first VRE session, B rated his confidence in his ability to ‘walk past pigeons in the street’ at 3.5; his parents rated his confidence as 1. By the end of the fourth VRE session his increased confidence rating was 5; his parent rating was 4.

The family reported that one week after the final VRE session, B was able to walk past pigeons in real life at the entrance to a shopping mall and was no longer afraid to sit near a window. Six weeks later, he was able to sit in a public square with pigeons without being constantly vigilant. At interview, B said he still had a fear of pigeons but that ‘it does not control me anymore’. He also reported being able to use the techniques he learned in the Blue Room in other situations - he had a much milder fear of dogs, and was able to use the relaxation techniques and coping statements if he met a dog while outside. At 6 months the effect was maintained with B still using the techniques he had learned in the VRE to control his anxiety. One year after treatment, B was able to control his anxiety without using the relaxation techniques. At 12–16 months post treatment, child B demonstrated reliable change on total SCAS scores for both parent and child report.

### Child F

Child F was aged 11 years and had a diagnosis of Asperger’s syndrome. He was referred by the local community mental health team and was receiving treatment for chronic anxiety. F had a phobia of crowded buses that was a result of a specific incident which had left him worried about something unexpected happening when he was on a bus. His VRE scene was as for child D.

F progressed through the stages of the treatment and was able to tolerate an increasing number of people on the virtual bus. However, in contrast to the experiences of other children, F found the VRE scenes did not lead him to feel present in the situation. His self-rating of confidence at getting on a bus was 4 at the beginning and end of the VRE sessions. Two weeks later, in real life, although he was able to walk to a bus stop, he was still unable to get on a bus. He was able to tell us that it was because he was ‘afraid of people acting strangely’. In parallel with participating in the treatment programme, his anxiety was exacerbated by transitions he was undergoing such as making decisions about his next school placement. At six months post treatment he was still unable to get on a bus. However, at 12–16 months follow up he had been able to ride on a public bus once, although with a great deal of family support.

## Discussion

This development study shows that CBT techniques delivered by a therapist in an immersive virtual reality environment can be a highly effective treatment for specific phobia/fear for some young people with ASD. Eight of the nine children improved in their ability to tackle their target situation and four children completely overcame their phobia. These improvements were maintained at 12–16 months after VRE treatment.

Nevertheless for two children, the approach was less successful. For child F it seemed that the scenario did not capture precisely enough the focus of his anticipatory anxiety. Furthermore, his level of general anxiety was high at the time of the VRE therapy as reflected in both self report and parent report anxiety scores on the SCAS. Child A, who also had high baseline SCAS scores, improved least of the children who responded to the treatment. This may indicate that very high initial anxiety levels might interfere with the treatment effect. Determining which young people may benefit most from the intervention requires further study. In future studies with larger groups of children it will be important to investigate impact of treatment on specific anxiety subtypes. Due to the exploratory nature of the current study and the small number of children included, we focussed on investigating the potential change in anxiety utilising the SCAS total score as a measure of overall level of anxiety. The total score on the SCAS is a composite of the scores across six subscales and potentially therefore captures types of anxiety that were not the target of our programme, thus providing us with a relatively conservative approach to determine the impact of the intervention. Despite this we were able to detect reliable change at 12–16 months for four children on the parent SCAS and four children on the child SCAS. Future research that is powered to explore putative change at the sub-scale level will provide us with more information.

Many children with autism spectrum disorder (ASD) show anxiety in response to particular settings. For children with ASD, their peers, parents and teachers, anxiety is problematic, as it causes distress, and has broader consequences, limiting opportunities to participate in activities and learning [41], [42]. In the current study, families reported that the positive management of anxiety generalised into other areas of the child’s life and activities.

The study adds to growing evidence that modified CBT can be effective for young people with ASD and high anxiety. CBT combined with immersive VRE has potential to be developed as a widely available treatment for anxiety related to specific fears and phobias in children with ASD. The addition of a VRE appears to offer many advantages over CBT alone or CBT with more traditional exposure therapy for this group.

Firstly, VRE offers a method of exposure that is particularly beneficial for therapists working with children with ASD. Since the aim is to keep the child’s anxiety at a low level in the VRE, the therapist is able to interact with the child while they are calm and attentive. The child is able to convey their inner state with minimal abstract language required through use of visual scales. This is particularly important for children with ASD who can find describing their inner world difficult [43]–[45]. They are also in a calm state and are able to focus on their thoughts and monitor the reactions in their bodies. This might not be the case if the child were being treated with real life exposure where anxiety levels might increase sharply on exposure. In that situation, as children with ASD can be slower to self-regulate emotions and return to baseline levels of arousal [46]–[47]; this might lead to persistent anxiety and treatment failure.

Secondly, VRE with CBT offers a greater degree of control over the stimuli than with CBT alone. The challenge of a VRE scene can be controlled, and the child can dictate the pace at which steps in the exposure proceed. The therapist and child are experiencing the scene at the same time and so have a common point of reference for therapeutic work, without the need for a great deal of verbal exchange. The scene can also be repeated with total consistency as many times as the child requires it. At all times the children feel that they are in control of the situation and know that nothing unexpected will happen. At the same time they learn they have control over their own anxiety level, through use of active techniques such as relaxation and breathing techniques, using positive coping statements. This seems to lead to a feeling of achievement and understanding of self coping techniques that is powerful enough to be translated to a feeling of confidence in the real life situation.

The third advantage is that VRE taps into a number of strengths and characteristics, and interests of those with ASD. There is evidence that children with ASD respond better to visual methods of learning [48] and learn well through the use of computers. Computers have been shown to lead to greater attention and motivation for learning in children with ASD than in a purely behavioural programme [49]. The VRE treatment has a clear structure, with repetition allowed at many stages to reinforce and consolidate learning and to help build confidence. Sensory input can be controlled in the VRE, e.g. adjusting noise levels and increasing sound gradually.

Finally, involving parents is an important element of successful CBT for those with ASD [14]. In our study parents valued being able to watch the CBT techniques being used with their children in the VRE, as they learned the techniques too. This gave them a clear understanding of how to keep exposure steps small and gradual, and equipped parents to try the same coaching techniques when in the real life situation with their child.

Importantly, several of the participants had similar phobia/fear topics such as buses or shopping. Mayes et al [11] in their study of phobia/fears in children with ASD also found recurring topics such as fear of toilets, mechanical objects, heights and weather. In the future, we will develop a ‘library’ of scenes involving common phobias/fears, which can then be adapted according to the needs of a particular child (e.g. placing their favourite food in a supermarket scene). This will reduce the time to make individualised scenes and increase the practical utility of the VRE treatment.

## Strengths and Limitations

This was a rigorously conducted study, which included children and families in which phobia/fear had a significant impact. Eight of the nine children were recruited through a research database. Whilst they had clear specific phobia/fear that led to an impact on everyday life, they were not being treated clinically for anxiety or phobias. In the future, the inclusion of a larger group of children and young people whose anxiety has led to referral to health services will be important to investigate treatment effects in these populations. The inclusion of a control group in future studies will be of importance; however, there is already good research evidence that CBT alone may not significantly improve specific phobia/fear in individuals with ASD [18].

In the future, this effective VRE/CBT treatment could be commissioned by health services as a treatment for specific phobia/fear in ASD. Clinical research assessments of children undergoing treatment in health services will be important, to investigate the treatment’s effectiveness further, and identify moderators and mediators of effect. It will be important to show that other therapists (of whatever background) can be trained to deliver the treatment with fidelity.

Although the aim of this study was to address difficulties associated with specific phobia, several of the scenes were of social situations e.g. public transport, a supermarket, a classroom. There may, therefore, be potential for this technology to be used to treat social anxieties associated with particular situations or specific triggers. Finally, this study only included children – we anticipate that the treatment would be effective in adults with ASD, and people with phobia/fear from the general population.

## Supporting Information

Protocol S1 **Trial Protocol.**
(DOC)

Checklist S1 **TREND Checklist.**
(DOC)
