# Supplementary material for: Reducing Specific Phobia/Fear in Young People with Autism Spectrum Disorders (ASDs) through a Virtual Reality Environment Intervention
Source: PLoS One. 2014 Jul 2;9(7):e100374. doi: 10.1371/journal.pone.0100374 (PMC4079659; doi:10.1371/journal.pone.0100374)
Supplement: Protocol S1 — Trial Protocol. (DOC) [file pone.0100374.s001.doc]

Protocol

Reducing Anxiety in Children with Autism Spectrum Disorder through Virtual Reality Environments

The aim of this study is to carry out a feasibility study of the use of a virtual reality environment, within a psychological treatment process, to aid reduction of anxiety in young people with autism spectrum disorder (ASD)

**Introduction**

Young people with ASD seem to be particularly at risk of developing anxiety, with as many as 42% meeting criteria for an anxiety disorder (White et al 2009). Anxiety may exacerbate the social impairments associated with ASD and may result in young people avoiding the situations they find stressful. This can also have a big impact on the families, caregivers and teachers of those on the spectrum.

Graduated exposure is identified as the key therapeutic mechanism in evidence-based treatments for phobias (Ollendick and King, 2006) but may require adaption for the particular characteristics of individuals with ASD. For example, graduated exposure may begin with imaginal desensitisation; however, individuals with ASD have difficulties with imagination, which means they can find producing and controlling imaginal scenes difficult.

One solution to this might be the use of virtual reality environments (VREs). VREs offer a powerful tool for training as participants become active members of a computer generated 3D virtual world. Participants can navigate through an environment (for example, a street or school) and interact with objects and people. Newly learned skills can be rehearsed and reinforced by role play in a safe and controlled environment. VREs have been used successfully in the wider population to treat fear of flying and heights (Rothbaum and Hodges,1999) and fear of public speaking (Slater et al, 2006). They have also been used successfully with people with ASD to improve various skills, for example social understanding (Mitchell et al, 2007), understanding facial expressions (Fabri et al, 2007), road safety and fire alarm procedures (Josman et al, 2008; Strickland et al, 2007).

Within this study, we want to explore the use of VREs as a therapeutic tool for young people with ASD, allowing them to develop coping strategies and increased confidence through the use of graduated exposure and strategy modelling by a therapist, in a controlled ‘real’-seeming environment. The information gathered will inform a future grant application to RfPB stream of NIHR funding for a pilot randomised trial of the VRE technology.

The research team have developed a working relationship with staff at the ‘Blue Room’ which is a state of the art VRE developed by a County Durham based company, Third Eye. The Blue Room uses images projected onto the walls and ceilings of a blue screened room. Participants are not required to wear a headset or goggles and can move around the room freely. Wallace and colleagues (2010) have carried out initial testing in the Blue Room and showed that children with ASD feel comfortable in the VRE, and feel they are ‘present’ in the scenarios depicted.

Within the Blue Room, an individualised environment can be created for each child. This might be photographic images of a real life scenario which has caused them anxiety, or an animated image of a similar situation.

While the child is in the Blue Room, we would also like to pilot a novel outcome measure so that it can be evaluated for use in a future randomised trial. Currently we can ask children about anxiety, but this may be difficult for them to report accurately or to be able to identify. In this study we would like to use a galvanic skin response monitor as a more direct measure of arousal. The galvanic skin response (GSR) monitor is worn on the wrist. It has been used successfully with young people with ASD and found acceptable to wear and gives an accurate measure of arousal (Picard, 2009). These measurements, as well as the questionnaires that will be administered, will allow an understanding of the relationship between arousal and emotional state.

As this is a development study, we are not testing hypotheses. However, it is expected that reported anxiety will be lower at the end of the intervention than at the start, and that measured arousal will be lower at the end of each session in the Blue Room than at the start of the session.

**Method**

***Participants***

The participants will be up to 20 children and young people with ASD, recruited through local Child and Adolescent Mental Health Services and through Dasl^n^e, the Newcastle University database of children with ASD living in the North East. The families on the Dasl^n^e database have consented to being approached about participating in future research studies.

Inclusion criteria: the child and parents are interested in undertaking a short experimental intervention to try to reduce a specific anxiety; aged between 8 and 14 years; a diagnosis of ASD/autism/Asperger syndrome; fluent verbal skills.

Exclusion Criteria: children with a co-morbid disorder e.g. depression, severe conduct disorder; children with severe generalised anxiety disorder; unable to travel to the VRE in Durham. As this is a development study and our budget will not cover translation costs, families have to be fluent in English.

***Measures***

Baseline characterisation

*Social Communication Questionnaire (SCQ)*

A parent completed 40 item questionnaire (yes/no questions) to describe the child’s ASD characteristics. It is the ‘gold standard’ questionnaire of its type and is used in ASD studies internationally.

Outcome measures

*Spence Children’s Anxiety Scale-parent version(SCAS-P) and child version (SCAS-C)*

The SCAS-C (Spence, 1998) was developed to assess anxiety symptoms in children in the general population. The SCAS-P (Spence,1998) is the parent version corresponding to the child version of the SCAS. The SCAS has 44 items on a 0 (never) to 3 (always) scale and comprises six subscales, including panic attack and agoraphobia (9 items), separation anxiety disorder (6 items), social phobia (6 items), physical injury fears (5 items), obsessive compulsive disorder (6 items) and generalized anxiety disorder (6 items). Six items are positively worded filler items (excluded from the parent version). The measure is widely used in ASD studies (Sofronoff et al, 2005; Chalfant et al, 2007). The SCAS shows high internal consistency, not only for the total scale, but also for each subscale (Spence,1998). The SCAS shows both convergent and divergent validity (Nauta et al, 2004). This questionnaire will be completed by both the parent and child at baseline and immediately after the intervention programme. We will also aim to repeat the questionnaires at 6 months post treatment and, if possible, to follow up at 12 months post treatment

The SCAS-P and SCAS-C will also be used as an initial screening measure to indicate where children may have severe and generalised anxiety and thus be excluded from the project.

*Galvanic Skin Response (GSR)*

GSR will be measured as a physiological indicator of arousal. This will be measured using a wireless device by Affectiva. The device is worn as a small wristband so it is relatively unobtrusive to the wearer. An individual’s baseline measure of arousal can be taken in less than 15 minutes. This provides a base point, against which any increase in arousal can be compared. The GSR will be worn by children for the baseline measurement prior to the Blue Room task, and then during the whole session in the Blue Room. Specific events can be ‘marked’ on the device providing an indication of any fluctuations in arousal in response to changes in the environment.

*Video recording*

Children will be video recorded during the Blue Room session and this will be relayed to the parents who will be outside the Blue Room, in an adjacent seating area. We will ask parents to rate the level of confidence shown by their child during the session using a visual five point analogue scale.

*Child’s anxiety and confidence rating*

At the beginning and end of each session, the child will rate how anxious they feel and how confident they feel at managing the situation that makes them anxious, using a visual five point analogue scale (similar to that given to parents).

*Qualitative information*

Semi-structured interviews will be carried out with the child and parent(s) within 6 weeks after the intervention, to examine their views of its appropriateness and helpfulness. These will be recorded and transcribed, and analysed using thematic analysis. Thematic analysis (Braun and Clark, 2006) is used in order to extrapolate the main themes and sub-themes that could be derived from the data. Such a form of analysis allows the researcher to retain some flexibility in decision making, whilst carefully identifying patterns of responses, resulting in a rich presentation of the issues discussed.

*Procedure*

*Recruitment and consent*

For recruitment from local CAMHS professionals, researchers will contact local teams and visit to outline the research study. The teams would then pass on information packs to families where the child met the inclusion criteria.

In addition, permission to approach members of the Dasl^n^e database will be sought by the researchers from the guardians of the database by submitting an application to their Research committee meeting. Potential participants screened for age and ability will be informed about the study by the Dasl^n^e coordinator. An information pack, containing both parent and child information sheets, will be sent with this letter. Potential participants will be asked to complete an Expression of Interest form and return it to the researchers in the SAE provided. Those who return this form will be contacted by researchers and a time arranged to meet with the family in their home. In this meeting the researcher will answer any questions or queries about the study and take informed consent. The baseline questionnaires (SCQ, SCAS-P and SCAS-C) will also be completed.

*Treatment Plan*

Preliminary Assessment Session (Session 1)

This will be a clinical assessment of the child’s anxiety in order to select a target which might be addressed therapeutically by virtual reality. The target is likely to be a specific phobia (e.g. of thunderstorms, supermarkets, hand dryers in public toilets) or possibly a recurring difficult social situation (such as reading classwork out loud). During this meeting we will explore with the child possible steps to full exposure. There will also be an initial discussion around how the child identifies feelings (e.g. how different parts of the body feel, what thoughts they have), introducing the concept of a ‘feeling thermometer’. This is a visual analogue scale used as a way of communicating level of anxiety.

It will be explained to families that there will be a gap between this assessment session and the anxiety treatment sessions, due to the need to photograph/animate the particular scenario chosen for the virtual reality sessions.

Treatment Sessions including Blue Room

Sessions 2-5 will take place at the virtual reality facility and will be carefully planned so that the child experiences a feeling of coping and mastery. For example, the psychologist present during the sessions will model and practice relaxation techniques and coping self statements before entering the Blue Room with the child. We will also be able to show them the readings from the GSR by session 3, to show that if anxiety levels go up then they will also come down. It is planned for each child to have two treatment sessions per visit, with a rest gap in between. If two sessions are enough to reduce anxiety substantially, then the VRE exposure will stop there with that particular child. Otherwise the child will return on another visit for sessions 4 and 5.

The final session (session 6) will be real life exposure to the situation we have been working with, where practicable, supported by the psychologist.

*Exit interviews*

Within one month of the final exposure session, the family will be visited again for a repeat of the Spence questionnaires and for the semi structured interview.

*Ethical considerations*

The treatment plan will be developed and overseen by Professor Helen McConachie, Consultant Clinical Psychologist in Child and Adolescent Mental Health. There will be close supervision of the psychology assistant running sessions 2-6. All staff involved with the project have enhanced CRBs.

Where a child is judged to have generalised anxiety of a level too severe for inclusion in the project, the research team will discuss with the family any appropriate clinical referral.

If a child participant in the project indicates a raised level of anxiety at the end of a treatment session,the psychologist will do further relaxation and cognitive work with them before they leave. However, this is judged to be unlikely, as the treatment programme is designed to proceed at the child’s pace and to create a feeling of mastery during gradual exposure.

An accessible information sheet will describe what will be involved if the potential participant agrees to take part in the study. Participants will be informed that they do not have to take part in the study if they do not want to. Participants will be informed that they are free to withdraw from the study at any time if they change their mind about participation, without any effect on other services they are receiving. If participants do decide to withdraw they will be asked if they would be happy for the researchers to include in the study any data collected up to that point.

With regards to confidentiality, data collected will be entered on a secure database at Newcastle University. Paper consent forms and any paper correspondence and data will be stored in locked filing cabinets within the swipe card protected Sir James Spence Institute in the Royal Victoria Infirmary, Newcastle.

Researchers will comply with Newcastle University lone worker procedures.

*Analyses*

For this development study, the main analyses will be of visual patterns of data, but with some estimation of effect size of change. Analysis will examine patterns in galvanic skin response data during sessions, and its relationship with anxiety and reported confidence before and after sessions. End point anxiety measured using the Spence Children’s Anxiety Scale for total score and subscales (particularly panic and agoraphobia, and physical injury fears) will be related to initial levels of anxiety. The feasibility and acceptability of the intervention approach will be analysed from the qualitative interviews, and will influence the next stage in developing a trial of VRE therapeutic intervention.

*Dissemination of the research findings*

The findings of this study will inform a grant application for a larger study. The findings will be published in a peer reviewed journal and presented at national conferences. The results will also be given to the Daphne Jackson Trust, from whom one of the researchers has obtained a Fellowship. We will also write to the research participants to let them know our findings, with one version for children and one for adults, and these summaries will be posted on the Dasl^n^e website.

*References*

Braun, V., & Clarke, V. (2006). Using thematic analysis in psychology. *Qualitative Research in Psychology,* 3, 77-101.

Chalfant, A.,Rapee, R., & Carroll, L. (2007). Treating anxiety disorders in children with high functioning autism spectrum disorders: A controlled trial. *Journal of Autism* *and Developmental Disorders*, 37, 1842-1857

Fabri, M., Elzouki, S.Y.A. & Moore, D. (2007) ‘Emotionally Expressive Avatars for Chatting, Learning and Therapeutic Intervention’, Proceedings of the 12th International Conference on Human-Computer Interaction (HCI International), July 22-27, 2007, Beijing, 4552, 275-285.

Josman, N., Milika Ben-Chaim, H., Friedrich, S. & Weiss, P.L. (2008) Effectiveness of Virtual Reality for Teaching Street-Crossing Skills to Children and Adolescents with Autism, *International Journal on Disability and Human Development* 7:49-56

Mitchell, P., Parsons, S. & Leonard, A. (2007) Using Virtual Environments for Teaching Social Understanding to Adolescents with Autistic Spectrum Disorders, *Journal of Autism and Developmental Disorders* 37:589-600

Nauta, M .H., Scholing, A.; Rapee, R.M.; Abbott, M., Spence S.H. and Waters, A . (2004) A parent-report measure of children's anxiety: psychometric properties and comparison with child-report in a clinic and normal sample. *Behaviour Research & Therapy*, **42**:. 813–839

Ollendick, T.H. & King, N.J (2004) Empirically supported treatments for children and adolescents: Advances towards evidence-based practice. In P.M. Barett & T.H. Ollendick (Eds.). *Handbook of interventions that work with children and adolescents*: *Prevention and treatment* (pp3-25) Chichester, England: Wiley

Picard, R.W.,(2009) Future Affective Technology for Autism and Emotion Communication’ *Philosophical Transactions of the Royal Society B: Biological Sciences*, .364: 3575-3584

Rothbaum, B.O. & Hodges, L.F. (1999) The Use of Virtual Reality Exposure in the Treatment of Anxiety Disorders, *Behaviour Modification* 23: 507-25

Slater, M., Pertaub, D., Barker, C. and Clark D. (2006) An Experimental Study on Fear of Public Speaking Using a Virtual Environment’ *CyberPsychology & Behavior* 9, (5), 627-633

Sofronoff, K., Attwood, T., & Hinton, S. (2005). A randomized controlled trial of CBT intervention for anxiety in children with Asperger syndrome. *Journal of Child* *Psychology and Psychiatry*, 46, 1152-1160.

Spence, H.S. (1998) A measure of anxiety symptoms among children. *Behaviour Research & Therapy*, 36: 545-566

Strickland, D.C., McAllister, D., Coles, C.D. & Osborne, S. (2007) An Evolution of Virtual Reality Training Designs for Children with Autism and Fetal Alcohol Spectrum Disorder’, *Topics in Language Disorders* 27: 226-241

Wallace, S., Parsons, S., Westbury, A., White, K, White, K and Bailey, A. (2010) Sense of Presence and Atypical Social Judgements in Immersive Virtual Environments: Responses of adolescents with Autism Spectrum Disorders’ *Autism* 14 (3) 199-213

White, S.W., Oswald, D., Ollendick, T. & Scahill, L. (2009) Anxiety in children and adolescents with autism spectrum disorders. *Clinical Psychology Review,* 29 (3), 216-229.
